# Identification of MiR-205 As a MicroRNA That Is Highly Expressed in Medullary Thymic Epithelial Cells

**DOI:** 10.1371/journal.pone.0135440

**Published:** 2015-08-13

**Authors:** Imran S. Khan, Chong Y. Park, Anastasia Mavropoulos, Nikki Shariat, Joshua L. Pollack, Andrea J. Barczak, David J. Erle, Michael T. McManus, Mark S. Anderson, Lukas T. Jeker

**Affiliations:** 1 UCSF Diabetes Center, University of California San Francisco, San Francisco, California, United States of America; 2 WM Keck Center for Noncoding RNAs, University of California San Francisco, San Francisco, California, United States of America; 3 Department of Medicine, University of California San Francisco, San Francisco, California, United States of America; 4 Department of Microbiology and Immunology, University of California San Francisco, San Francisco, California, United States of America; 5 Department of Pathology, University of California San Francisco, San Francisco, California, United States of America; Universidade de Sao Paulo, BRAZIL

## Abstract

Thymic epithelial cells (TECs) support T cell development in the thymus. Cortical thymic epithelial cells (cTECs) facilitate positive selection of developing thymocytes whereas medullary thymic epithelial cells (mTECs) facilitate the deletion of self-reactive thymocytes in order to prevent autoimmunity. The mTEC compartment is highly dynamic with continuous maturation and turnover, but the genetic regulation of these processes remains poorly understood. MicroRNAs (miRNAs) are important regulators of TEC genetic programs since miRNA-deficient TECs are severely defective. However, the individual miRNAs important for TEC maintenance and function and their mechanisms of action remain unknown. Here, we demonstrate that miR-205 is highly and preferentially expressed in mTECs during both thymic ontogeny and in the postnatal thymus. This distinct expression is suggestive of functional importance for TEC biology. Genetic ablation of miR-205 in TECs, however, neither revealed a role for miR-205 in TEC function during homeostatic conditions nor during recovery from thymic stress conditions. Thus, despite its distinct expression, miR-205 on its own is largely dispensable for mTEC biology.

## Introduction

Thymic epithelial cells (TECs) are critical mediators of T cell development in the thymus. Cortical thymic epithelial cells (cTECs) facilitate positive selection as thymocytes rearrange and assemble T cell receptors (TCRs) capable of recognizing self-MHC [1]. Positively selected thymocytes migrate to the medulla to undergo negative selection by medullary thymic epithelial cells (mTECs) [1, 2]. To prevent autoimmunity, mTECs eliminate self-reactive thymocytes from the developing T cell pool by displaying a repertoire of tissue-specific antigens (TSAs) whose expression is normally limited to peripheral tissues [3–6]. This ectopic expression of TSAs is largely dependent on autoimmune regulator (Aire), which is expressed in a mature subset of mTECs [7–9]. Patients and mice with defects in *Aire* develop multi-organ autoimmune disease, which emphasizes the importance of TSA expression in mTECs as a means to promote central T cell tolerance [7, 10–12].

Several groups have recently shown that mTECs and cTECs represent a highly dynamic cell population with continuous cycling and turnover in the postnatal thymus [13–19]. However, the precise regulation of these processes and their impact on thymic function remain largely unknown. While thymocytes represent one of the best genetically characterized cell types, genetic programs in TECs are poorly understood [20]. Thus, further work is necessary to understand the regulation of gene expression necessary for maintaining homeostasis within the TEC compartments.

MicroRNAs (miRNAs) are ~22 nucleotide “noncoding” RNAs that mediate post-transcriptional repression of genes in a sequence-dependent manner [21, 22]. Primary miRNA transcripts are processed by the DROSHA/DGCR8 complex to generate ~60-80nt hairpin precursor miRNAs [23]. These hairpins are further processed in the cytoplasm by Dicer to produce mature miRNAs. Mature miRNAs mediate gene repression through complementary base-pairing mostly within the 3’-untranslated region (UTR) of target mRNAs. Each miRNA can target hundreds of mRNAs, and each mRNA can in turn be regulated by many miRNAs [22, 23]. Thus, miRNAs represent key regulators of gene networks and can be exploited to discover novel pathways.

Recent work by our group and others has shown that complete miRNA-deficiency in TECs causes a severe disruption of thymic architecture and function *in vivo* which leads to the breakdown of central tolerance [24–27]. While these studies demonstrate the importance of miRNAs as a class of genes, the individual miRNAs controlling gene expression and thus TEC function remain largely unknown. Identifying the specific miRNAs which are important for TEC biology promises to uncover novel genetic networks that are important for establishing and maintaining central tolerance. However, there is currently no consensus on which miRNAs are expressed in TECs [26, 28, 29]. Evolutionarily conserved expression of a set of miRNAs in murine and human TECs is indicative that those miRNAs are important, but experimental evidence testing the function of individual miRNAs is largely missing [26]. Aire appears to control the expression of a subset of miRNAs but different groups reported discordant results depending on whether *Aire* was ablated genetically [26], or whether it was manipulated *in vitro* using siRNA transfection in a mTEC cell line [28]. Consistent with the absence of a consensus on TEC-specific miRNA expression it remains controversial which TSA are regulated by miRNAs, if at all [26, 29]. In summary, although it is well accepted that miRNAs as a class of posttranscriptional regulators are important for TEC biology, the role of individual miRNAs is unclear. However, a better understanding of the regulation of the thymic microenvironment might be exploited for therapeutic interventions aiming to promote or ablate TEC function [30, 31].

Here, we profiled miRNA expression in murine TECs and demonstrate a distinct expression of miR-205 in mTECs. Using a multimodal approach involving gene expression arrays, real time gene expression analysis, *in situ* hybridization as well as a murine knock-in reporter allele we characterize its expression during thymic ontogeny and in the postnatal thymus. In addition, for functional analysis, we utilized a conditional knockout allele to ablate miR-205 in TEC lineages. However, despite strong and distinct miR-205 expression suggestive of a particular function, mice lacking miR-205 in TECs showed normal thymic architecture and T cell development. Furthermore, we did not find evidence of autoimmunity or defects in central tolerance. Finally, we were unable to detect phenotypic defects in miR-205-deficient TECs under both homeostatic and thymic stress conditions.

In summary, miR-205 is strongly expressed in mTECs but its function in the postnatal thymus and its functional relevance in mTEC biology remain unknown. Future studies are needed to test whether other miRNAs, e.g. the miR-200 family, are functionally redundant with miR-205 or if miR-205 plays a non-redundant role for a specific function not examined in our experiments.

## Results and Discussion

### miR-205 is expressed in medullary thymic epithelial cells

**Fig 1:**
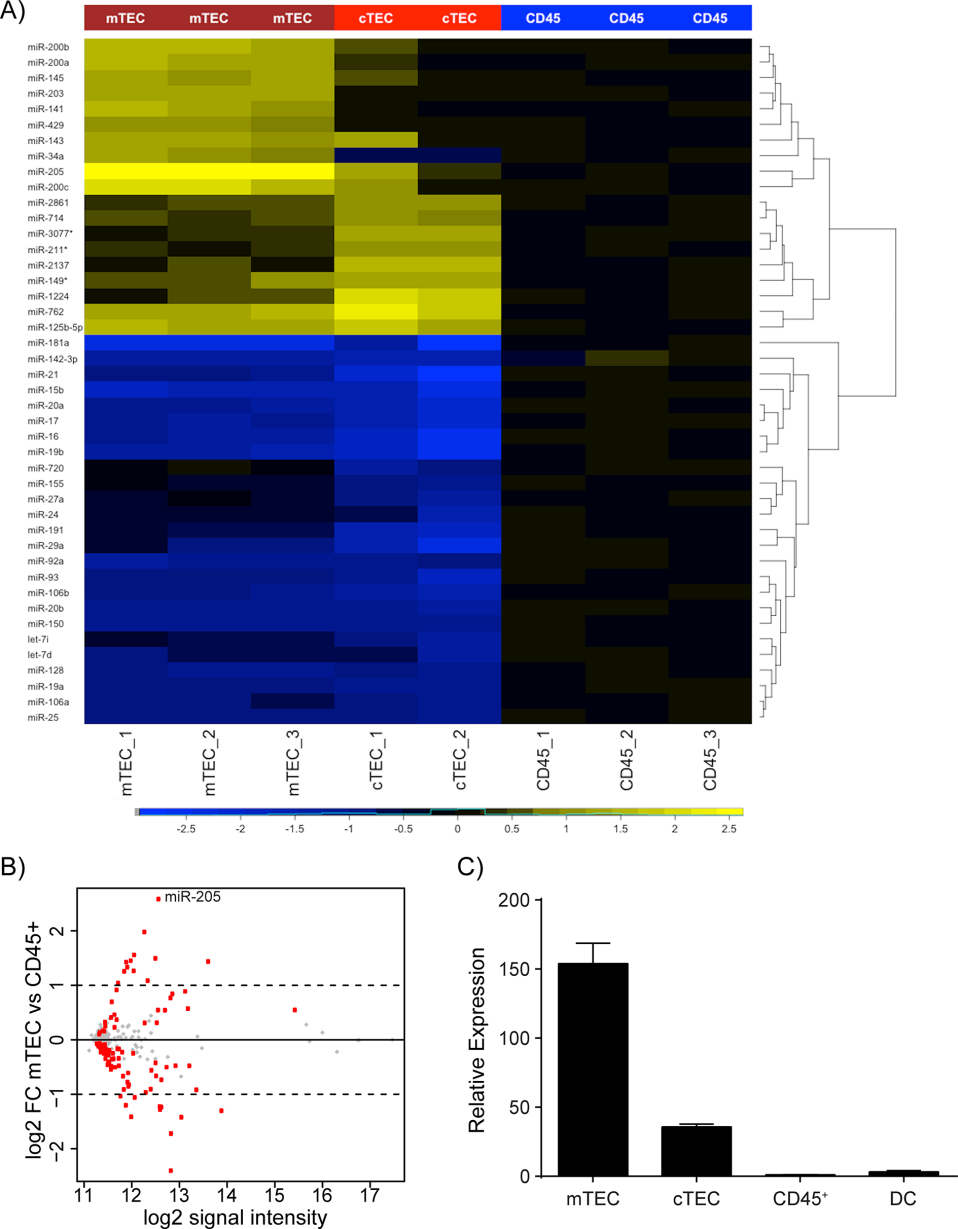
TEC miRNA profiling identifies miR-205 as highly expressed in mTECs. **(A)** Thymic subsets were purified from 4–5 week old *NOD* wildtype mice for miRNA profiling by microarray analysis. The heatmap depicts the union of differentially expressed miRNAs from any comparison (FDR <0.05) with an absolute log2 fold change >1 relative to the signal intensity of CD45^+^ cells. **(B)** Plot depicts average log2 fold change (FC) between mTEC vs CD45^+^ cells on the *y*-axis and average log2 signal intensity across all samples on the *x*-axis. Red dots indicate genes that are differentially expressed in mTECs vs CD45^+^ cells with an FDR <0.05. **(C)** Thymic stromal subsets were FACS-purified from 4–6 week old *B6* wildtype mice to confirm the expression of miR-205 in mTECs by qPCR analysis. All reactions were standardized to sno202 and then normalized to CD45^+^ cells with error bars depicting mean ±SD.

In order to identify miRNAs differentially expressed in medullary thymic epithelial cells (mTECs), we purified thymic cell subsets from adult mice by flow cytometric cell sorting. We sorted mTECs, cortical thymic epithelial cells (cTECs), and CD45^+^ cells as three distinct populations and prepared RNA for microarray analysis (S1 Fig). The CD45^+^ thymocytes were included as a reference population in order to identify TEC-specific miRNAs. These microarray data revealed a distinct miRNA signature for each cell type while also demonstrating a high degree of reproducibility amongst the replicate samples (Fig 1A). Specifically, we found all five members of the miR-200 family (miR-141, miR-200a, miR-200b, miR-200c and miR-429) to be preferentially expressed in TEC compared to hematopoietic cells. In addition, miR-143, miR-145, miR-203 and miR-205 were among the top differentially expressed miRNAs. In contrast, miRNAs characteristic of lymphocytes were underrepresented in TECs compared to CD45^+^ cells. These results are in good agreement with a previously published miRNA expression profiling study which found the same relative differential expression of all these miRNAs [26]. Of note, expression of the miR-200 family, miR-203 and miR-205 are characteristic of epithelial cells in which they are functionally relevant during differentiation and to maintain epithelial identity [32–34]. Thus, our results suggest that multiple miRNAs might be involved in TEC differentiation and the induction and maintenance of epithelial identity of TECs. Cell-type specific genetic ablation of individual miRNAs is a powerful tool to investigate the functional contribution of individual miRNAs in a given cell type. However, although we and others have in some cases successfully used this approach, miRNA-ablation often does not result in overt phenotypic consequences [35]. Nevertheless, to begin the tedious process of functionally characterizing individual miRNAs in TECs we chose to focus on miR-205 because of the following reasons: a) its high signal intensity in mTECs, b) its differential expression in mTECs when compared to CD45^+^ cells and cTECs (Fig 1A–1C), c) no other known miRNA shares the same seed sequence, d) miR-205-deficient mice are among the few miRNA-deficient mice that display neonatal lethality indicating that miR-205 has non-redundant functions at least in certain cells [36, 37]. First, to validate the microarray data we analyzed purified thymic cell subsets by qPCR. miR-205 expression was confirmed to be strongly expressed in mTECs, showed intermediate expression in cTECs, and was expressed at much lower levels in CD45^+^ and thymic dendritic cells (DC) (Fig 1C and S2 Fig).

**Fig 2:**
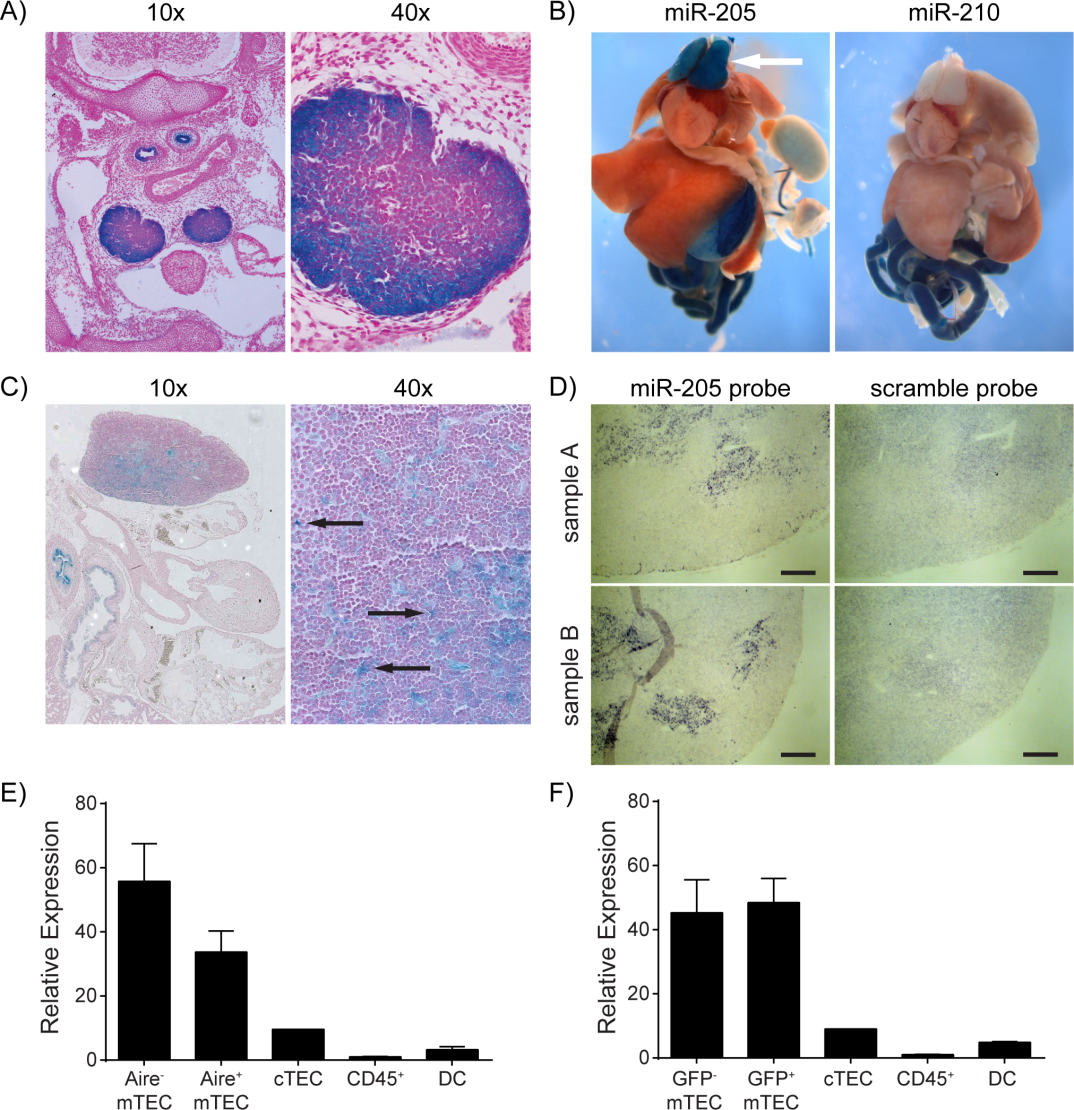
miR-205 is expressed during thymic ontogeny and maintained in the adult thymus. **(A)** X-gal staining was performed on transverse sections of e14.5 *miR-205* ^lacZ^ embryos to identify patterns of miR-205 transcription. **(B)** Whole-mount X-gal staining was performed on a dissected e18.5 *miR-205* ^lacZ^ embryo (left) and positive lacZ reporter activity was observed in the thymus (arrow). A dissected and stained *miR-210* ^lacZ^ embryo (right) is shown as a negative control for lacZ activity in the thymus. These images have been published previously at http://rna.keck.ucsf.edu/sites/rna.keck.ucsf.edu/files/205_E18.5_051510_24.jpg. **(C)** Thymic sections from an e18.5 *miR-205* ^lacZ^ embryo were cut and then stained with X-gal. Arrows indicate positive lacZ reporter activity in a subset of cells in the thymus. **(D)** *in situ* hybridization for miR-205 was performed on frozen thymic sections from 6–8 week old B6 wildtype mice. Serial sections were hybridized using either a miR-205 probe or a scramble probe. Image pairs from two samples are shown. Scale bars = 200 μm. **(E-F)** Sorted thymic subsets from either *Aire* ^+/+^ **(E)** or *Aire* ^-/-^ **(F)** mice were analyzed by qPCR for miR-205 expression. Both genotypes carried the Aire-GFP (*Adig*) allele to facilitate the sorting of Aire^+^/GFP^+^ and Aire^-^/GFP^-^ mTEC subsets. Reactions were standardized to sno202 and then normalized to CD45^+^ cells with error bars depicting mean ±SD.

While microarrays and qPCR are useful to screen, confirm and quantitate miRNA expression in pooled cells the purification disrupts the natural tissue architecture. Therefore, to characterize the physiologic temporal and spatial expression pattern of miR-205 we utilized a conditional knockout allele in which a promoter-less lacZ reporter and loxP sites were targeted into the miR-205 locus (*miR-205* ^lacZ^) (S3 Fig) [38]. While this conditional allele can be used to ablate miR-205 in a tissue-specific manner, its promoter-less lacZ reporter allows for monitoring of the transcriptional activity of the endogenous miR-205 locus. We performed whole-mount X-gal staining of *miR-205* ^lacZ^ embryos and observed strong lacZ activity in the thymus as early as embryonic day e14.5 (Fig 2A) as previously reported [36]. Besides the thymic tissue, the bronchial and esophageal epithelium also stained positive. At e18.5 we observed prominent lacZ staining of both thymic lobes while the other major organs remained lacZ negative. Control lacZ knock-in reporter mice where lacZ expression reports transcriptional activity of the miR-210 locus did not show any thymic staining (Fig 2B). X-gal staining of tissue sections from e18.5 *miR-205* ^lacZ^ embryos showed positive lacZ activity within a scattered subset of cells within the developing thymus (Fig 2C). The limited number and the pattern of cells showing positive lacZ activity suggested miR-205 expression in the stromal compartment of the thymus rather than thymocytes. Since transcriptional activity does not necessarily correlate with mature miRNA expression [39] we performed *in situ* hybridization for mature miR-205 in wildtype mice to both validate these lacZ reporter findings and to characterize miR-205 expression in the adult thymus. *In situ* hybridization experiments confirmed a staining pattern consistent with expression throughout the thymic medulla and suggested that mTECs which express miR-205 do not show any distinct anatomical localization with respect to the cortex or the corticomedullary junction (Fig 2D).

Since several subsets of mTECs have been defined, we further analyzed miR-205 expression within the mTEC compartment. mTEC differentiation begins as mTEC^lo^ (MHC II^lo^ Aire^-^) cells and at later stages mTECs upregulate MHC II and Aire. This leads to a transition through an mTEC^hi^ (MHC II^hi^, Aire^-^) stage and eventually results in Aire^+^ (MHC II^hi^, Aire^+^) cells [9, 13, 40]. To isolate these subsets we took advantage of an Aire-GFP (*Adig*) reporter allele to purify both immature Aire^-^ and mature Aire^+^ mTEC subsets from *Aire* ^+/+^ mice and performed qPCR analysis for miR-205 [41]. Our results demonstrate that miR-205 is highly expressed in both mTEC subsets with a slight enrichment in the immature Aire^-^ mTECs (Fig 2E). Differential miRNA expression between Aire^-^ and Aire^+^ mTEC was recently reported for miRNAs other than miR-205 [26], and transfection of anti-*Aire* siRNA changed miRNA expression in cell culture [28]. Therefore we tested if Aire influences miR-205 expression. When we used the *Adig* reporter to purify GFP^+^ and GFP^-^ mTECs from *Aire* ^*-/-*^ mice, we found that although miR-205 was still highly expressed in both mTEC subsets, its expression was more comparable between the two subsets (Fig 2F). Recent work has also suggested that Aire is required in mTECs in order for them to complete their differentiation program [42–44]. Our results therefore suggest that Aire may partially repress miR-205 expression or that Aire is required for terminal differentiation of mTECs which then indirectly results in somewhat reduced miR-205 expression. Taken together, our results demonstrate that miR-205 is highly expressed in mTECs during both thymic ontogeny and in the postnatal thymus.

### TEC-specific ablation of miR-205

**Fig 3:**
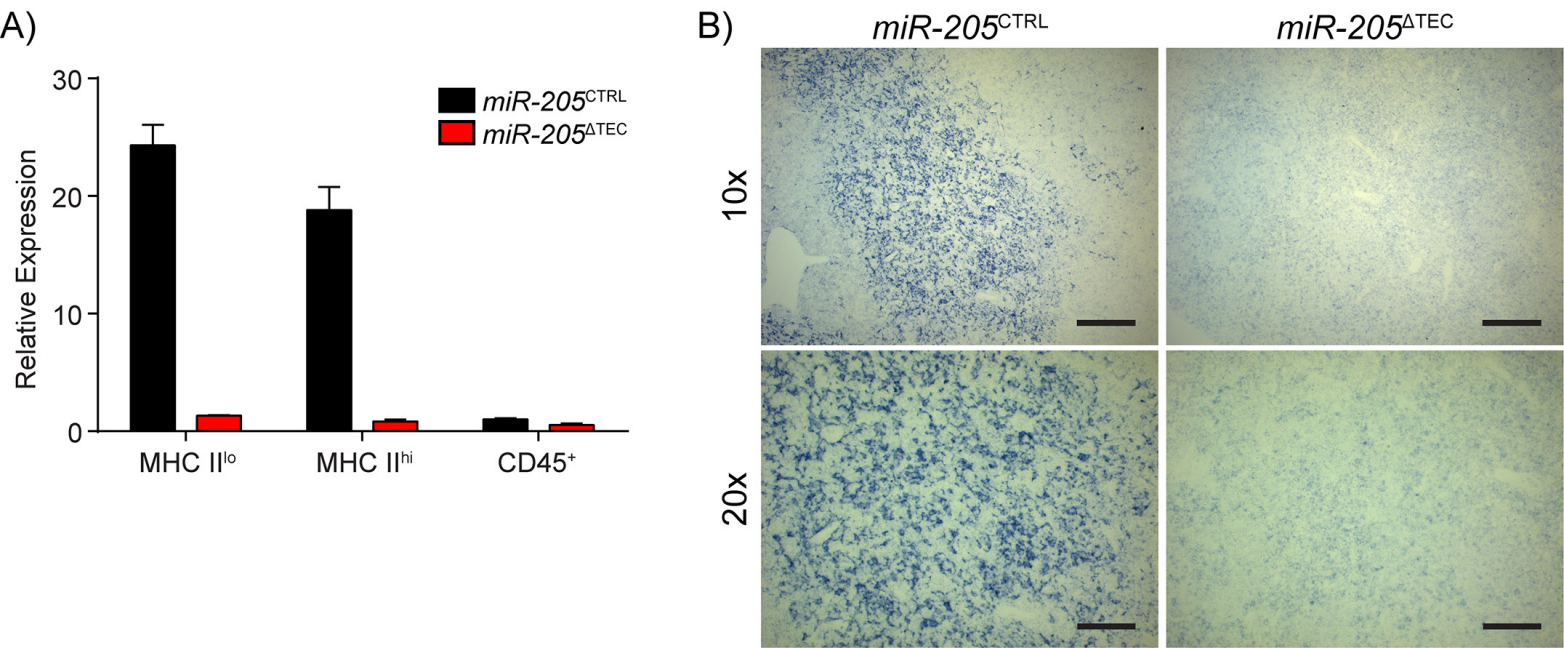
Validation of miR-205 ablation in TECs. **(A)** mTECs from 4–6 week old *miR-205* ^CTRL^ and *miR-205* ^ΔTEC^ mice were sorted to analyze miR-205 expression by qPCR. Reactions were normalized to sno202 and normalized to CD45^+^ cells. Values depict mean ±SD. Data is representative of two independent experiments. **(B)** *in situ* hybridizations were performed using a miR-205 probe on frozen thymic sections from 4–6 week old *miR-205* ^CTRL^ and *miR-205* ^ΔTEC^ mice to confirm the uniform deletion of miR-205 in mTECs. Scale bars = 200 μm (top), and 100 μm (bottom).

To study the role of miR-205 in TEC function we crossed *miR-205* ^lacZ^ mice to a Rosa26-Flp strain to excise the lacZ/neomycin cassette (S3 Fig) [45]. Removing the lacZ/neomycin cassette helps to ensure physiologic expression of the conditionally targeted gene [46]. The Flp deleter allele was then bred out. We next utilized *FoxN1-Cre* knock-in mice, which express Cre recombinase in all TECs without disrupting FoxN1 function [47], to specifically inactivate miR-205 in TECs (*miR-205* ^ΔTEC^) (S3 Fig). Purification of mTECs from both *miR-205* ^CTRL^ and *miR-205* ^ΔTEC^ demonstrated both the proper expression of miR-205 in *miR-205* ^CTRL^ mice as well as its efficient ablation in *miR-205* ^ΔTEC^ (Fig 3A). In parallel, we performed *in situ* hybridization on thymi from these mice to demonstrate uniform ablation of mature miR-205 in *miR-205* ^ΔTEC^ mice (Fig 3B).

### miR-205 deficiency in TECs does not perturb thymic function under homeostasis

**Fig 4:**
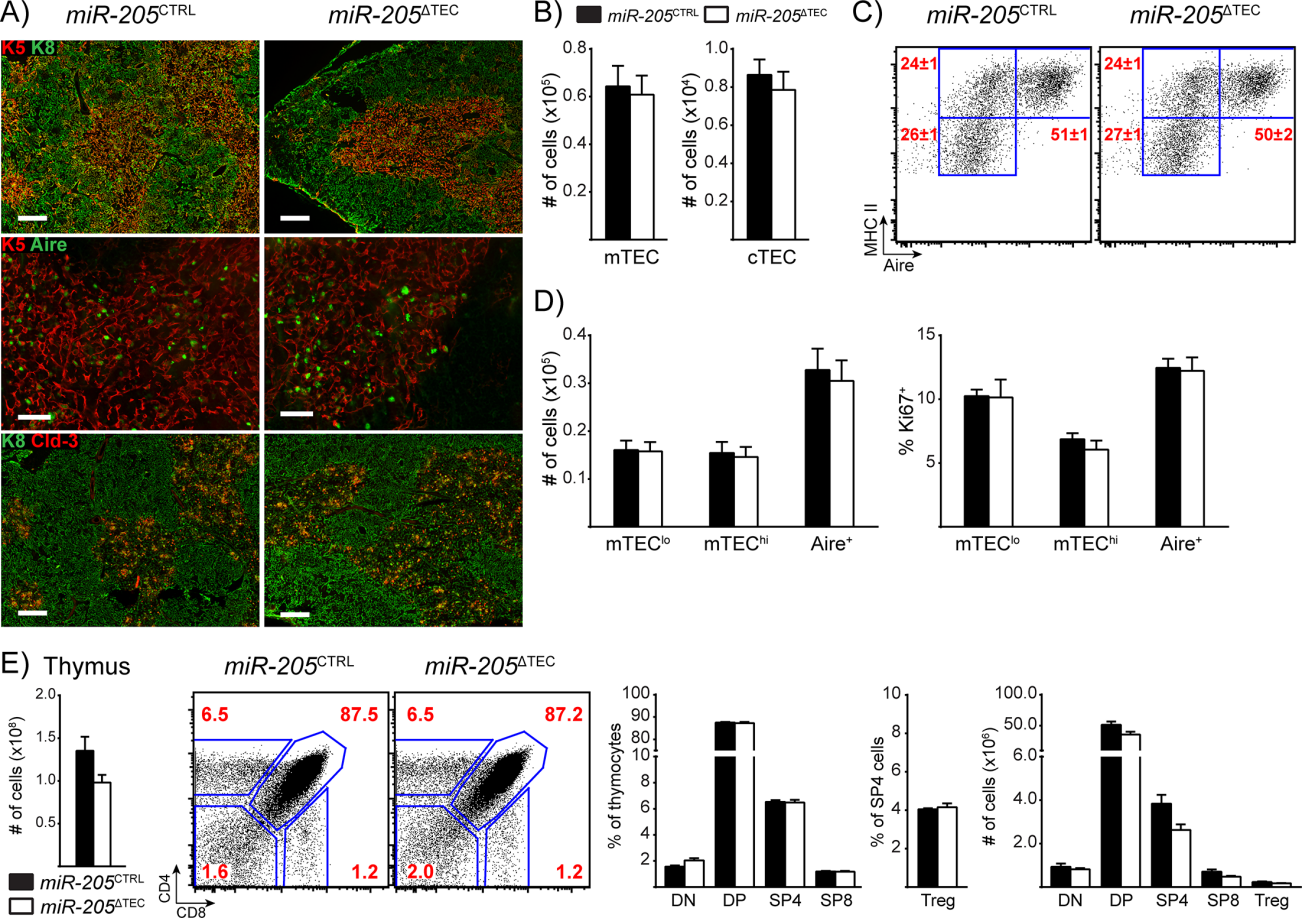
miR-205 deficient thymi appear phenotypically normal under homeostatic conditions. **(A)** Frozen thymic sections from 6-week old *miR-205* ^ΔTEC^ and littermate control mice were assessed for expression of keratin-5 (K5, red), keratin-8 (K8, green), claudin-3 (Cld-3, red), and Aire (green). Top = K5 and K8, scale bars = 200 μm. Middle = K5 and Aire, scale bars = 50 μm. Bottom = K8 and Cld-3, scale bars = 200 μm. **(B)** Enumeration of total mTEC and cTEC cellularity in 5-month old mice by flow cytometry. cTECs were defined as CD45^-^, EpCAM^+^, Ly51^+^, MHC II^+^ events. mTECs were defined as CD45^-^, EpCAM^+^, Ly51^-^, MHC II^+^ events. **(C)** Subset composition was assessed by flow cytometry of mTECs as defined in **(B)**. **(D)** Quantification of total TEC cellularity and assessment of the proliferation marker Ki67 for the mTEC subsets shown in **(C)**. mTEC subsets were defined as mTEC^lo^ (MHC II^low^, Aire^-^), mTEC^hi^ (MHC II^hi^, Aire^-^), and Aire^+^ (MHC II^hi^, Aire^+^). Data in **(B-D)** are shown as mean ±SEM of 8 samples per group and representative of at least two independent experiments. **(E)** Total thymic cellularity from 4-month old mice was assessed by flow cytometry. Plots show thymocyte subsets: CD4^-^CD8^-^ double negative (DN), CD4^+^CD8^+^ double positive (DP), CD4^+^ single positive (SP), and CD8^+^ SP thymocytes. Relative frequencies are shown as a proportion of all thymocytes with the exception of Treg cells, which are shown as a proportion of CD4^+^ SP thymocytes. Data are shown as mean ±SEM of 3 samples and are representative of at least 3 independent experiments. White bars in **(B-E)** indicate *miR-205* ^ΔTEC^ mice, black bars indicate littermate controls.

To understand the physiologic role of miR-205 in TECs during homeostasis, we first analyzed unmanipulated adult *miR-205* ^CTRL^ and *miR-205* ^ΔTEC^ mice. Immunostaining of thymi revealed similar patterns of the surrogate markers for the cortex (keratin-8; K8) and medulla (keratin-5; K5), indicating that the overall corticomedullary architecture of the thymus was preserved in *miR-205* ^ΔTEC^ mice (Fig 4A). Staining for Aire was also comparable between the two genotypes, which suggested that mTEC maturation was largely intact in the absence of miR-205 in TECs (Fig 4A).

Previous work has shown that miR-205 can target *Zeb2* and thereby play a role in regulating the epithelial-to-mesenchymal transition (EMT) [32, 48, 49]. EMT is an important physiologic process during embryonic development and wound repair [50], but its role in TEC biology remains unknown. *Zeb1* and *Zeb2* are transcription factors which drive EMT progression by downregulating “epithelial” genes such as E-cadherin, claudins and occludins while upregulating “mesenchymal” genes such as N-cadherin, vimentin and fibronectin [50, 51]. We therefore hypothesized that miR-205-deficiency might lead to a decreased epithelial signature. Based on a recent report suggesting that Aire^+^ mTECs are derived from a unique claudin-3,4^+^ lineage of mTECs [52] we stained for claudin-3 on thymic sections from adult *miR-205* ^CTRL^ and *miR-205* ^ΔTEC^ mice. However, as in the case for K5 and K8 we did not observe any difference between the two genotypes (Fig 4A).

To quantify TEC subsets from miR-205-deficient and-sufficient mice we performed flow cytometry on TECs. Consistent with the histology, we neither observed any significant differences in mTEC nor cTEC cellularity (Fig 4B). We used MHC II and Aire to further characterize the mTEC compartment and found the immature mTEC^lo^ (MHC II^lo^ Aire^-^), intermediate mTEC^hi^ (MHC II^hi^, Aire^-^) and mature Aire^+^ (MHC II^hi^, Aire^+^) mTEC subsets to be comparable in both proportion and cell number between *miR-205* ^CTRL^ and *miR-205* ^ΔTEC^ mice (Fig 4C–4D). Furthermore, there were no differences in the proportion of mTEC subsets which expressed the proliferation marker Ki67 (Fig 4D). Taken together, these results indicate that miR-205 deficient thymi showed no changes in the development, maintenance, or maturation of TECs.

Next, we characterized the impact of miR-205-deficiency on thymocyte maturation. We did not observe any significant changes in total thymic cellularity between *miR-205* ^CTRL^ and *miR-205* ^ΔTEC^ mice (Fig 4E). Similarly, we did not observe any significant differences in the relative frequencies or absolute numbers of CD4^-^CD8^-^ double negative (DN), CD4^+^CD8^+^ double positive (DP), CD4^+^ single positive (SP), CD8^+^ SP thymocytes or CD4^+^Foxp3^+^ Treg cells (Fig 4E). Overall, thymocyte maturation appeared unchanged, suggesting that miR-205 deficient TECs are sufficient to support T cell development.

### Central and peripheral tolerance is maintained with miR-205 deficiency in TECs

**Fig 5:**
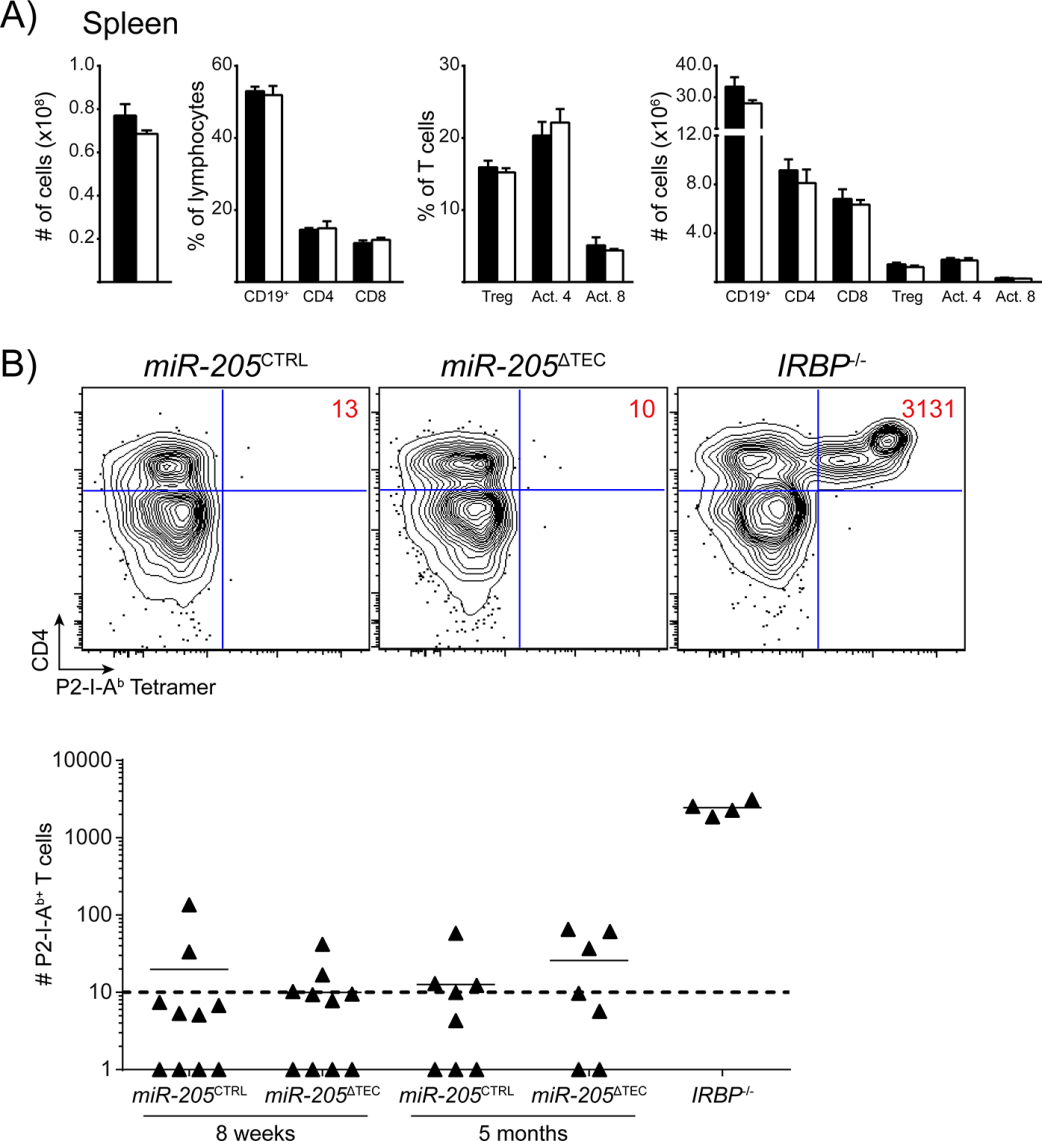
Central and peripheral tolerance is maintained in miR-205-deficient mice. **(A)** Total splenic cellularity from 4-month old mice. Indicated lymphocyte subsets are shown as a proportion of all splenocytes with the exception of Treg cells (CD4^+^ CD25^+^ Foxp3^+^) and activated-memory phenotype T cells (CD44^hi^ CD62L^lo^), which are shown as a proportion of their respective T cell populations. Splenocyte data are shown as mean ± SEM of 3 samples per group, and are representative of at least 3 independent experiments. White bars indicate *miR-205* ^ΔTEC^ mice, black bars indicate littermate controls. **(B)** Mice were immunized with P2 peptide and then harvested 10 days later and analyzed by flow cytometry following a tetramer pulldown assay. Plots are pre-gated on DAPI^-^, NK1.1^-^, CD11b^-^, CD11c^-^, F4/80^-^, B220^-^, CD3^+^ events. Absolute numbers of P2-specific cells are inset within the flow cytometry plots and plotted below. Tetramer data are pooled from 8–10 samples per group from two independent experiments. *IRBP* ^*-/-*^ mice were included as a positive control for immunization and tetramer pulldown.

We next sought to determine whether miR-205 deficiency in the thymus led to any changes in peripheral lymphocytes. We analyzed splenocytes from adult mice but did not detect any significant changes in the total number of splenocytes between *miR-205* ^CTRL^ and *miR-205* ^ΔTEC^ mice (Fig 5A). The relative frequency and absolute number of CD19^+^ cells, CD4^+^ T cells and CD8^+^ T cells was also comparable between the two genotypes (Fig 5A). Further analysis of T cell subsets showed no difference in Treg cells (CD25^+^ Foxp3^+^) and in T cells with an activated-memory (CD44^hi^ CD62L^lo^) phenotype (Fig 5A). Together, these results suggest that peripheral T cell homeostasis is maintained in *miR-205* ^ΔTEC^ mice.

Despite the largely intact thymic architecture, miR-205-deficiency could still result in aberrant thymocyte selection. Given the predominantly medullary expression of miR-205 a defect in thymocyte selection would most likely affect negative selection. Specifically, we hypothesized that self-reactive T cells could escape thymic deletion if miR-205-deficiency affected antigen processing or presentation. Importantly, absence of all miRNAs in TECs does not result in overt autoimmune disease and escaping self-reactive T cells remain in check most likely due to intact peripheral tolerance mechanisms [24]. To test for escaped self-reactive T cells within the polyclonal T cell repertoire, we utilized the previously reported immunization-based approach to expand and detect Aire-dependent autoreactive T cells in the periphery of *miR-205* ^ΔTEC^ mice [53]. T cells specific for the self-antigen IRBP are efficiently deleted in the thymus of *Aire*-sufficient hosts but in *Aire*-deficient mice or in mice lacking all miRNAs in TEC these cells can escape thymic deletion and, when expanded through immunization, provoke autoimmune uveitis [6, 24, 53]. Autoreactive CD4^+^ IRBP-specific cells can be detected in the periphery of *Aire* ^*-/-*^ mice through the use of a peptide-MHC class II tetramer, P2-I-A^b^ [24, 53]. To determine whether T cells escaped thymic deletion in *miR-205* ^ΔTEC^ mice, we immunized mice with an MHC II binding IRBP (P2) peptide to expand cells for detection in the periphery. Ten days following immunization, we pooled lymph nodes and spleen to enumerate total numbers of CD4^+^ P2-I-A^b^-specific T cells by flow cytometry. We did not detect any differences in the expansion of IRBP-specific T cells in either 8-week old or 5-month old *miR-205* ^ΔTEC^ mice when compared to controls (Fig 5B). Consistent with these findings we could not detect signs of spontaneous autoimmunity by hematoxylin and eosin (H&E) staining of various organs from aged *miR-205* ^ΔTEC^ mice (data not shown). Thus, we concluded that under homeostatic conditions, *miR-205* ^ΔTEC^ mice do not exhibit overt defects in central T cell tolerance.

### Thymic stress conditions do not reveal a role for miR-205 in TECs

Organisms lacking a specific miRNA often do not present overt phenotypic consequences under homeostatic conditions [35, 54–56]. Several reasons may account for this: i) miRNAs are often redundant but even ablation of multiple miRNAs of the same miRNA family does not necessarily result in obvious defects [57] ii) many miRNAs act as fine tuners of gene expression rather than molecular switches. Therefore, it has been proposed that the regulatory role of miRNAs is to buffer changes in gene expression during critical periods of physiological stress [58, 59]. Indeed, we reported previously that mice lacking miR-17-92 in Treg are phenotypically normal under homeostatic conditions. In contrast, miR-17-92-deficient Treg are severely impaired in their ability to control the immune response following the induction of experimental autoimmune encephalitis. As a result disease is much more severe with miR-17-92-deficient Treg than in control mice with wildtype Treg [60]. Therefore, we used two different thymic stress models in an attempt to reveal a TEC phenotype in *miR-205* ^ΔTEC^ mice.

**Fig 6:**
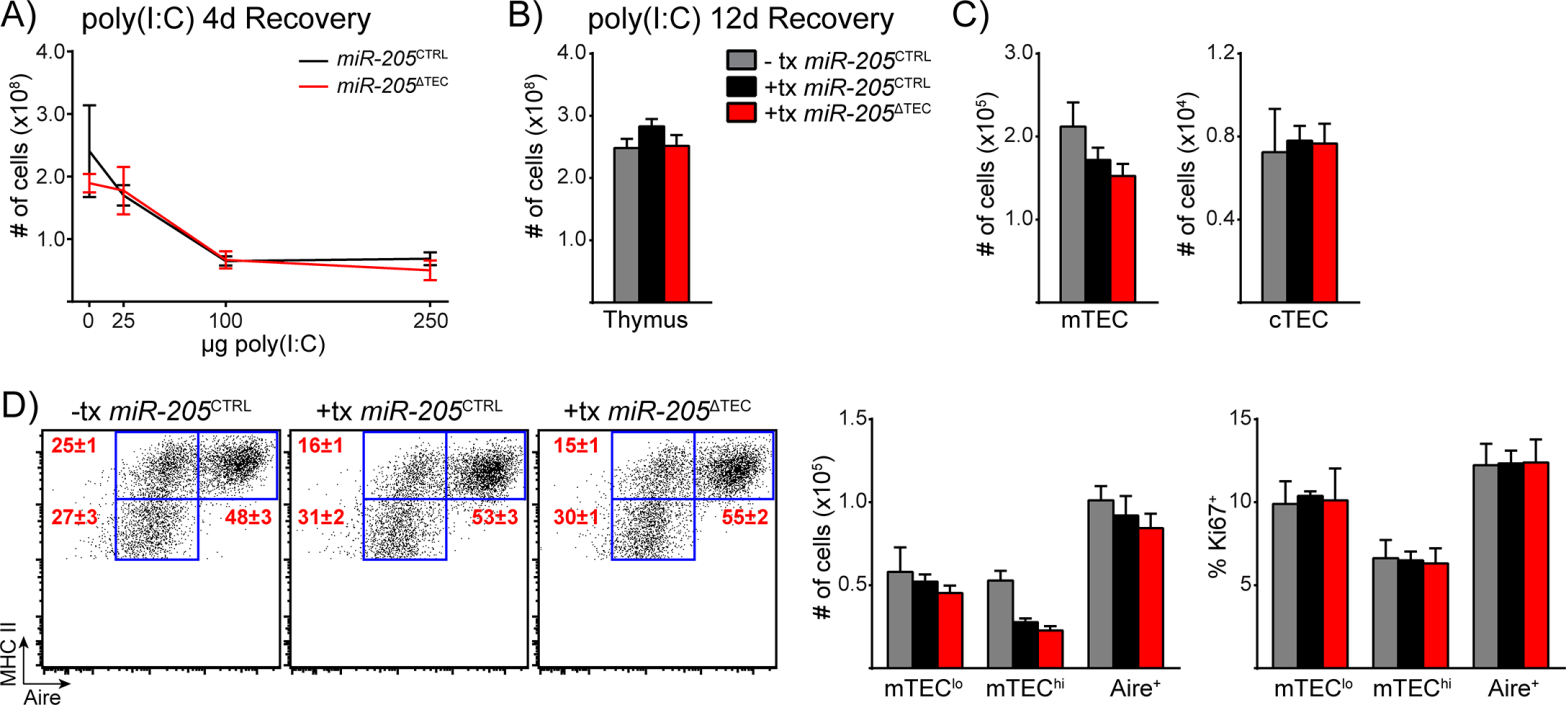
miR-205 deficient TECs show comparable sensitivity and recovery potential to poly(I:C) mediated thymic involution. **(A)** 4-week old mice were treated with varying doses of poly(I:C) at day (-3) and day (0) before being harvested at day 4 of their recovery for enumeration of total thymic cellularity. **(B)** Mice were treated with 250μg of poly(I:C) as conducted in **(A)** and then harvested at 12 days of recovery to enumerate total thymic cellularity. **(C)** Enumeration of total mTEC and cTEC cellularity in 4-week old mice shown in **(B)**. cTECs were defined as CD45^-^, EpCAM^+^, Ly51^+^, MHC II^+^ events. mTECs were defined as CD45^-^, EpCAM^+^, Ly51^-^, MHC II^+^ events. **(D)** Subset composition was assessed by flow cytometry of mTECs as defined in **(C)**. Quantification of total TEC cellularity and assessment of the proliferation marker Ki67 for the mTEC subsets shown on the left. Data in **(B-D)** are shown as mean ±SEM of 7 samples per group and are representative of at least two independent experiments. Gray bars in **(B-E)** indicate untreated *miR-205* ^CTRL^ mice, gray bars indicate poly(I:C) treated *miR-205* ^CTRL^ mice, and red bars indicate poly(I:C) treated*miR-205* ^ΔTEC^ mice.

The thymus involutes in response to various insults including hormones, infection, irradiation and inflammatory cytokines [16, 61–65], and recent work using TEC ablation models has shown that both cTECs and mTECs have a remarkable ability to recover from thymic injury [16, 17, 19]. Given the reports describing miR-205 as a regulator of cell cycle and proliferation [66–69], we hypothesized that TECs from *miR-205* ^ΔTEC^ mice would be impaired in their ability to recover from thymic damage. Systemic administration of polyinosinic-polycytidylic acid (poly(I:C)) demonstrated that miR-29a-deficiency lowers the threshold for the thymus to undergo involution in response to poly(I:C) [25]. Previous work showed substantial thymic involution 4 days after systemic administration of poly(I:C) along with significant thymic recovery by the 12-day timepoint [61]. In accordance with these studies we administered systemic poly(I:C) to *miR-205* ^ΔTEC^ mice and littermate controls and analyzed mice at both the 4-day and 12-day recovery timepoints. While we observed a substantial degree of thymic involution at 4-day timepoint following poly(I:C) administration, we failed to uncover any difference between the two groups (Fig 6A). To analyze the recovery from thymic injury we analyzed mice at the 12-day recovery timepoint following administration of the high-dose poly(I:C). Although total thymic cellularity had returned to baseline at this time, the mTEC compartment was still undergoing recovery as indicated by the lower cellularity in the mTEC^hi^ subset (Fig 6B–6D). However, again the relative and absolute composition of TEC subsets and their proliferative capacity did not differ between *miR-205* ^ΔTEC^ and control mice which suggested that miR-205 does not play a role in TECs during recovery from poly(I:C)-induced thymic involution.

**Fig 7:**
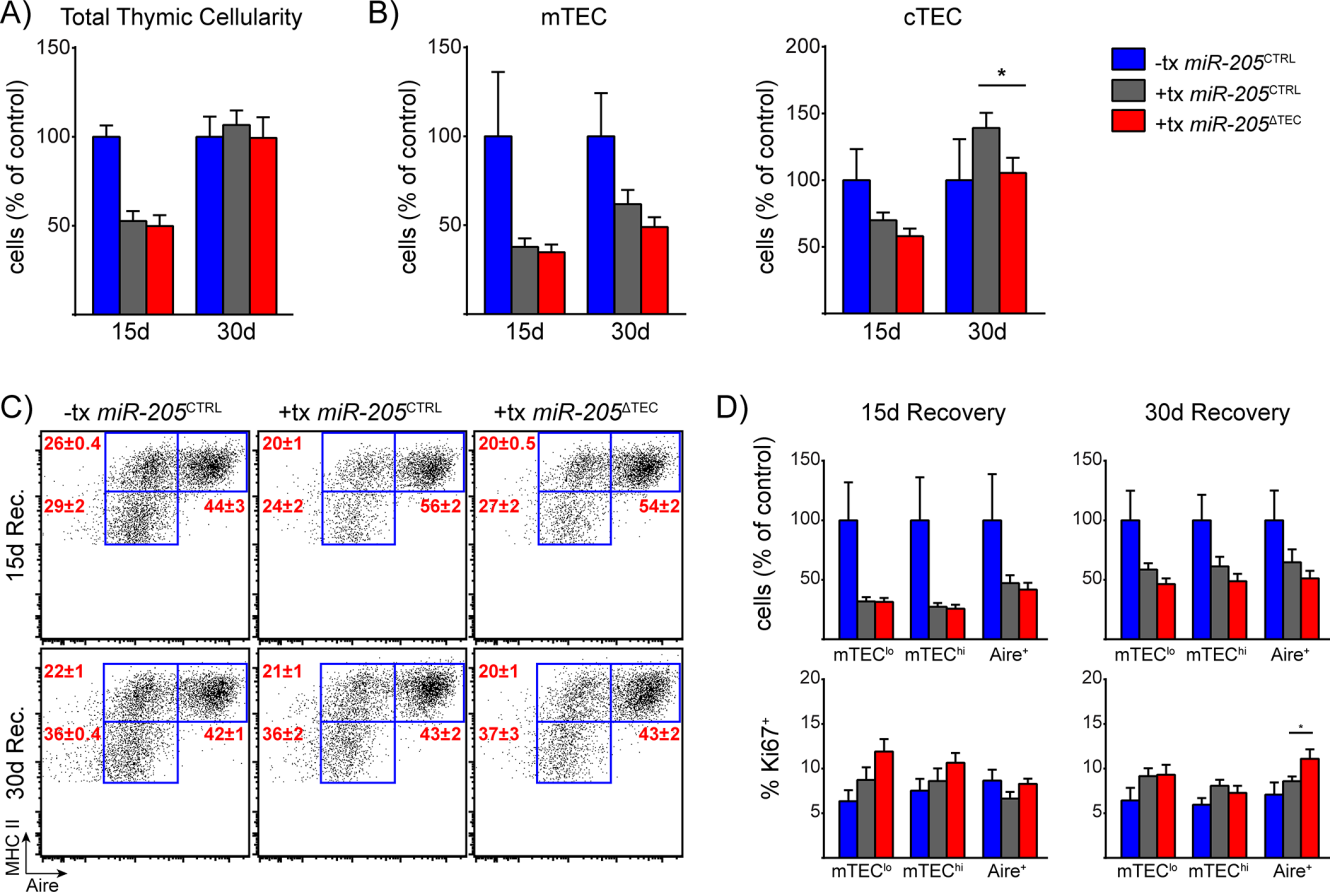
Radiation-induced thymic stress does not reveal a role for miR-205 in TECs. **(A)** 6-week old mice were exposed to sub-lethal total body irradiation and then harvested after either 15 or 30 days of recovery to enumerate total thymic cellularity. **(B)** Enumeration of total mTEC and cTEC cellularity from mice shown in **(A)**. mTECs were defined as CD45^-^, EpCAM^+^, Ly51^-^, MHC II^+^ events. cTECs were defined as CD45^-^, EpCAM^+^, Ly51^+^, MHC II^+^ events. **(C)** Subset composition of mTECs was assessed by flow cytometry of mTECs as defined in **(B)**. **(D)** Quantification of mTEC cellularity and assessment of the proliferation marker Ki67 for the mTEC subsets shown in **(C)**. Data in **(A-D)** are shown as mean ±SEM of 10 samples per group pooled from two independent experiments. Total cellularity plotted as percent of untreated *miR-205* ^CTRL^ mice to allow for direct comparison between the two timepoints. Blue bars indicate untreated *miR-205* ^CTRL^ mice, gray bars indicate treated *miR-205* ^CTRL^ mice, and red bars indicate treated *miR-205* ^ΔTEC^ mice. * denotes p≤0.05, Student’s *t*-test.

As a second independent thymic stress model, we chose to administer sub-lethal total body irradiation (SL-TBI) to *miR-205* ^CTRL^ and *miR-205* ^ΔTEC^ mice. Irradiation of the thymus causes thymic involution by promoting both thymocyte apoptosis as well as direct damage to the TEC compartment [62, 70, 71]. This was an important consideration in choosing this model because many models of thymic stress are driven by the depletion of developing thymocytes [16, 61, 72, 73], and the impact on the TEC compartment is likely secondary to the withdrawal of cross-talk interactions between developing thymocytes and the stromal compartment [16, 74]. After exposing mice to SL-TBI we harvested their thymi for analysis at two timepoints of recovery. Total thymocyte and TEC cellularity were equally depleted in both genotypes at 15-days post-irradiation when compared to untreated control thymi (Fig 7A and 7B). While total thymocyte cellularity of both irradiated genotypes had equally returned to baseline by 30-days post-irradiation, mTEC cellularity was still reduced in both irradiated genotypes compared to untreated control mice (Fig 7A and 7B). While mTEC cellularity was comparable between *miR-205* ^CTRL^ and *miR-205* ^ΔTEC^ mice at 15-day and 30-day timepoints, cTEC cellularity was slightly reduced in the *miR-205* ^ΔTEC^ mice at the 30-day timepoint (Fig 7B). All mTEC subsets were similar in frequency and total cell number between the two genotypes (Fig 7C and 7D). Of note, we observed a mildly increased proportion of Ki67^+^ Aire^+^ cells in *miR-205* ^ΔTEC^ compared to control mice (Fig 7D). The slightly decreased cTEC numbers at 30-days post-irradiation and the mildly increased frequency of proliferating Aire^+^ cells do not seem to bear a functional relevance on the overall ability of miR-205 deficient TECs to recover from SL-TBI mediated injury. Taken together, the results indicate that miR-205 is largely dispensable in TECs for recovery from either poly(I:C)- or SL-TBI-mediated thymic insult.

In summary, we have demonstrated that miR-205 is strongly and preferentially expressed in mTEC and to a lesser degree in cTEC. However, despite the suggestive expression pattern we were not able to conclusively define a function for miR-205 in TEC biology. There are many possible explanations for our inability to detect a phenotype in *miR-205* ^ΔTEC^ mice and hence uncover the role of miR-205 in mTECs. miR-205 is expressed in many epithelial tissues including the skin and stomach and is best characterized as an epithelial miRNA [33, 36, 75]. It is often co-expressed with several other “epithelial” miRNAs such as miR-203 and the miR-200 family [32, 33, 48]. While miR-205 does not have any known miRNA family members with conserved seed sequences, it remains possible that several of its co-expressed epithelial miRNAs could functionally compensate for it in the context of miR-205 deficiency. For example, although miR-205 has been implicated as a regulator of EMT through its ability to target *ZEB2*, the same studies showed an even greater functional relationship between members of the miR-200 family and EMT progression than with miR-205 [32, 48]. Indeed, miR-203 and all five members of the miR-200 family are co-expressed in mTECs which raises the question whether miR-205 and these other “epithelial” miRNAs co-regulate genes to ensure epithelial identity of TECs. Thus, it would be interesting to investigate whether ablation of multiple of these “epithelial” miRNAs will result in a TEC defect. Such a study would be challenging using a classical breeding approach but perhaps multiplexed miRNA targeting using genome editing could help overcome this obstacle. It also remains possible that the function of miR-205 in TECs is to regulate early developmental steps during thymic ontogeny, and hence our studies in the postnatal thymus were not sufficient to interrogate this role. A recently published study characterized miR-205 transcription throughout embryonic development by performing X-gal staining on *miR-205* ^lacZ^ embryos [36]. Of note, the authors were able to detect lacZ activity in the pharyngeal pouches at e11.5 and e12.5 [36]. This observation is of note because the earliest expression of FoxN1 correlates with these timepoints during thymic development [76, 77]. If the critical function of miR-205 was limited to this early period of development, *FoxN1-Cre*-mediated ablation of miR-205 might have occurred beyond the timepoint necessary to reveal the consequence of miR-205-deficiency in TECs. Alternatively, *miR-205* ^ΔTEC^ mice could have recovered from any transient defects during thymic development such that the postnatal thymus is phenotypically normal. However, in the event that miR-205 is only required during thymic development, it remains unclear why mTECs maintain such a high level of miR-205 expression in adult mice. Therefore, the most likely explanation seems to be functional redundancy with other miRNAs. Future studies ablating miR-205 in combination with other miRNAs might reveal its function in TEC biology although even combined ablation of entire miRNA families does not necessarily result in easily detectable defects [57].

### Concluding Remarks

In summary, we performed miRNA profiling of thymic stromal cells and identified miR-205 as a candidate miRNA with preferential expression in mTECs. We show here that miR-205 is highly expressed in mTEC populations during both embryonic development and in the postnatal thymus. To characterize the role of miR-205 in mTECs, we utilized a miR-205 conditional allele in combination with *FoxN1-Cre* to target the ablation of miR-205 to TEC lineages. We were unable to detect a phenotype in miR-205 deficient TECs in adult mice, and both thymocyte development and peripheral lymphocyte homeostasis appeared comparable between *miR-205* ^ΔTEC^ mice and controls. Immunization with an Aire-dependent self-antigen failed to reveal a breakdown in central tolerance, and *miR-205* ^ΔTEC^ mice did not show any other signs of overt autoimmunity. Finally, thymic stress models were unable to suggest a role for miR-205 in TECs as *miR-205* ^ΔTEC^ mice showed similar recovery to littermate controls following either poly(I:C) or SL-TBI mediated thymic involution. Thus, the function of miR-205 in the postnatal thymus remains elusive.

## Materials and Methods

### Mice

*FoxN1-Cre* knock-in mice were kindly provided by N. Manley [47]. Targeted *miR-205* (*miR-205* ^lacZ^) mice have been described previously [38]. To generate conditional knockout mice *miR-205* ^lacZ/+^ mice were crossed to a previously described Rosa-Flp allele to excise the lacZ/neomycin cassette [45]. After confirming the deletion of these two elements, we out-crossed the Rosa-Flp allele and then backcrossed *miR-205* ^fl/+^ mice to the C57BL/6J background for 3 generations. At this point *miR-205* ^fl/+^ mice were crossed with *B6*.*FoxN1-Cre* mice for experimental analysis. Throughout this study *miR-205* ^ΔTEC^ represents *FoxN1-Cre* ^*+*^ *miR-205* ^*fl/fl*^ mice and littermate controls are *FoxN1-Cre* ^*+*^ *miR-205* ^*fl/+*^ mice and all *FoxN1-Cre* ^*-*^ mice. *IRBP* ^-/-^, Aire-GFP (*Adig)*, and *Aire* ^-/-^ mice have been described previously [6, 7, 41]. Mice were housed and bred under specific-pathogen free conditions at the University of California, San Francisco (UCSF) Animal Barrier Facility. Animal experiments were approved by the UCSF Institutional Animal Care and Use Committee (IACUC) for this study (approval number AN091519).

### Thymic Stress Models

For poly(I:C)-induced thymic involution, High Molecular Weight poly(I:C) (Invivogen) was reconstituted in sterile saline according to the manufacturer’s instructions. Mice were treated intraperitoneally on day (-3) and day 0 and then harvested at the indicated recovery timepoints. For sub-lethal total body irradiation (SL-TBI) experiments, mice were exposed to a single 550 cGy dose of radiation on day 0 and allowed to recover without hematopoietic rescue until they were analyzed at the designated timepoints.

### Flow Cytometry

Thymic stromal cells were isolated as described previously [41, 78]. Briefly, thymi were minced with razor blades and digested with DNase I and Liberase TM (Roche) before gradient centrifugation with Percoll PLUS (GE Healthcare). Enriched stromal cells were first incubated with the Fc-receptor blocking antibody 2.4G2 and then stained with the indicated surface marker antibodies (BioLegend). For lymphocyte staining, all surface marker antibodies were obtained from BioLegend. For intracellular staining, cells were processed using the Foxp3 Staining Buffer Set and stained with anti-Foxp3-APC (eBiosciences), anti-Ki67-PE (BD Biosciences), or anti-Aire-A647 (eBiosciences). All data were collected using a BD LSR II flow cytometer and analyzed with either FloJo software (TreeStar) or FACS Diva (BD Biosciences). Cell sorting for microarray and qPCR analyses was performed using a BD FACS Aria III cell sorter.

### RNA Isolation

Total RNA was extracted from FACS-sorted samples using TRIzol (Invitrogen) according to the manufacturer’s instructions.

### Quantitative PCR

RNA was extracted as described above. As described previously [79], reverse transcriptase reactions were performed using the Applied Biosystems TaqMan MicroRNA RT kit and quantitative PCR reactions were performed using the Applied Biosystems TaqMan miRNA assay system. All reactions were normalized to sno202.

### Microarray Analysis

Thymic subsets were FACS-purified from 4-week old NOD wildtype mice. Thymi from 10–12 female mice were pooled together for stromal cell isolation, and RNA was extracted as described above. Sample preparation, labeling, and array hybridizations were performed according to standard protocols from the UCSF Shared Microarray Core Facilities and Agilent Technologies (http://www.arrays.ucsf.edu and http://www.agilent.com). Total RNA quality was assessed using a Pico Chip on an Agilent 2100 Bioanalyzer (Agilent Technologies, Palo Alto, CA). RNA was labeled with Cy3-CTP using the miRCURY LNA microRNA power labeling kit (Exiqon, Inc, Woburn, MA), according to manufacturer’s protocol. Labeled RNA was hybridized to Agilent custom UCSF miRNA v3.6 multi-species 8x15K Ink-jet arrays (Agilent). Hybridizations were performed for 16 hrs, according to the manufacturers protocol (Agilent). Arrays were scanned using the Agilent microarray scanner (Agilent) and raw signal intensities were extracted with Feature Extraction v10.1 software (Agilent). Data are available from Gene Expression Omnibus: accession number GSE68674.

### X-gal Staining

For staining of embryos, *miR-205* ^lacZ^ embryos were harvested and fixed with 4% paraformaldehyde and 0.2% glutaraldehyde as described previously [36, 38]. For larger e18.5 embryos, internal organs were dissected out for additional fixation and permeabilized in 0.02% NP40, 0.01% sodium deoxycholate, and 2mM PBS for one hour prior to staining. Overnight X-gal staining was performed at room temperature and embryos were then fixed in 2% paraformaldehyde and stored in 70% ethanol.

### *In Situ* Hybridization

*In situ* hybridization was performed on frozen thymic sections. At harvest, thymi were fixed for 2 hours in 4% paraformaldehyde and equilibrated for 7 hours in 30% sucrose. We used double DIG labeled LNA probes against either miR-205 or a scramble control (Exiqon). We followed the manufacturer’s instructions and hybridized the probes overnight at 57°C with the following modifications: Post-hybridization stringency washes: 2x SSC for 60’ at 57°C, 1x SSC for 10’ at RT, 0.5x SSC for 10’ at RT, 0.1x SSC for 45’ at 57°C. Tissues were then blocked with 1% goat serum in 0.1% PBS-Tween-20 (PBST) for 2 hours before overnight incubation with a 1:5000 dilution of anti-DIG-AP antibody (Roche) at 4°C. Following antibody incubation and overnight washes in PBST, alkaline phosphatase activity was detected using an NBT/BCIP solution (Roche). Slides were visualized using either a Zeiss Apotome or a Zeiss AxioImager brightfield microscope.

### Histology and Immunofluorescence

Thymi were harvested and embedded in Optimal Cutting Temperature (OCT) media (Tissue-Tek). 8μm frozen thymic sections were fixed in 100% acetone, blocked in 10% goat serum, and then stained for keratin-5 (Abcam), keratin-8 (Abcam), claudin-3 (Invitrogen), or Aire (eBiosciences). Secondary antibodies were purchased from Invitrogen. Immunofluorescent staining was visualized using a Zeiss Apotome widefield microscope.

### Immunization and Tetramer Analysis

Mice were immunized with 100 μg of IRBP P2 peptide (amino acids 271–290) emulsified in Complete Freund’s Adjuvant (CFA) as described previously [53]. Tetramer analysis was performed on pooled lymph nodes and spleen harvested from treated mice 10 days after immunization. P2-I-A^b^ tetramer (Interphotoreceptor retinol binding protein 3, amino acids 294–306) was generated by the NIH Tetramer Core Facility, and tetramer staining was performed as described previously [53, 80]. Briefly, cells were stained with tetramer for 1 hour at room temperature before enrichment for tetramer^+^ cells using anti-APC microbeads and MACS columns (Miltenyi Biotech). Positively-selected cells were stained with antibodies for flow cytometry, and counting beads (Invitrogen) were used to enumerate the absolute number of tetramer^+^ cells.

### Statistical Analysis

Statistical analysis was performed using Prism 6.0 (Graphpad). Mann-Whitney testing was performed for tetramer analysis. Student’s *t*-test was performed for TEC and lymphocyte analyses. * denotes p≤0.05, ** denotes p≤0.01 and *** denotes p≤0.001.

## Supporting Information

S1 Fig Gating strategy for FACS-purification of thymic subsets for microarray analysis. Thymic subsets were purified from 4–5 week old *NOD* wildtype mice for miRNA profiling by microarray analysis. cTECs were defined as CD45^-^, EpCAM^+^, MHC II^+^, Ly51^+^ events. mTECs were defined as CD45^-^, EpCAM^+^, MHC II^+^, Ly51^-^ events. CD45+ cells were defined as EpCAM^-^, CD45^+^ events.
(TIF)

S2 Fig Quantitative RT-PCR for miR-205 expression in medullary thymic epithelial cells. Thymic stromal subsets were purified by flow cytometry from 4–6 week old *C57BL/6J* wildtype mice to confirm the expression of miR-205 in mTECs by qPCR analysis. Amplification plots are shown for miR-205 and sno202 (internal reference gene) in mTECs and CD45^+^ cells. The amplification threshold is indicated in green and the threshold cycle for each probe and cell population is indicated in parentheses.
(TIF)

S3 Fig Overview of miR-205 conditional knockout targeting construct. The endogenous *miR-205* locus was targeted with a construct containing both a promoter-less lacZ reporter as well as a neomycin cassette. To generate conditional knockout mice, targeted mice were crossed to Rosa26-Flp mice (*miR-205* ^fl/fl^), and then bred to *FoxN1-Cre* mice to ablate miR-205 in TECs (*miR-205* ^ΔTEC^).
(TIF)
