# Supplementary figures and images for: Identification of MiR-205 As a MicroRNA That Is Highly Expressed in Medullary Thymic Epithelial Cells
Source: PLoS One. 2015 Aug 13;10(8):e0135440. doi: 10.1371/journal.pone.0135440 (PMC4535774; doi:10.1371/journal.pone.0135440)

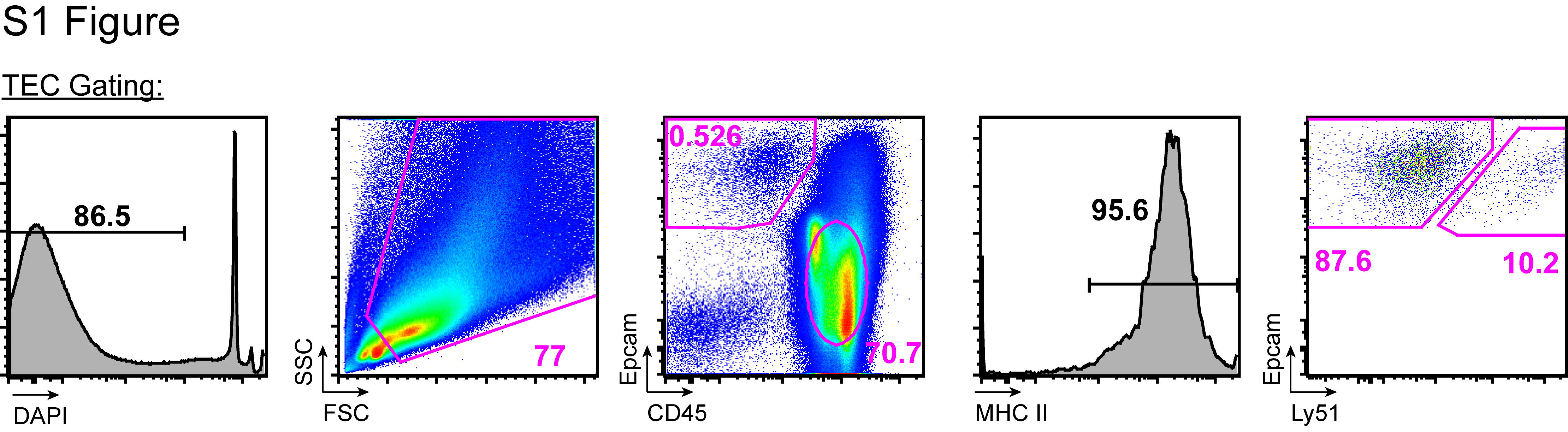

Supplement: S1 Fig — Thymic subsets were purified from 4–5 week old NOD wildtype mice for miRNA profiling by microarray analysis. cTECs were defined as CD45-, EpCAM+, MHC II+, Ly51+ events. mTECs were defined as CD45-, EpCAM+, MHC II+, Ly51- events. CD45+ cells were defined as EpCAM-, CD45+ events. (TIF) [file pone.0135440.s001.tif]

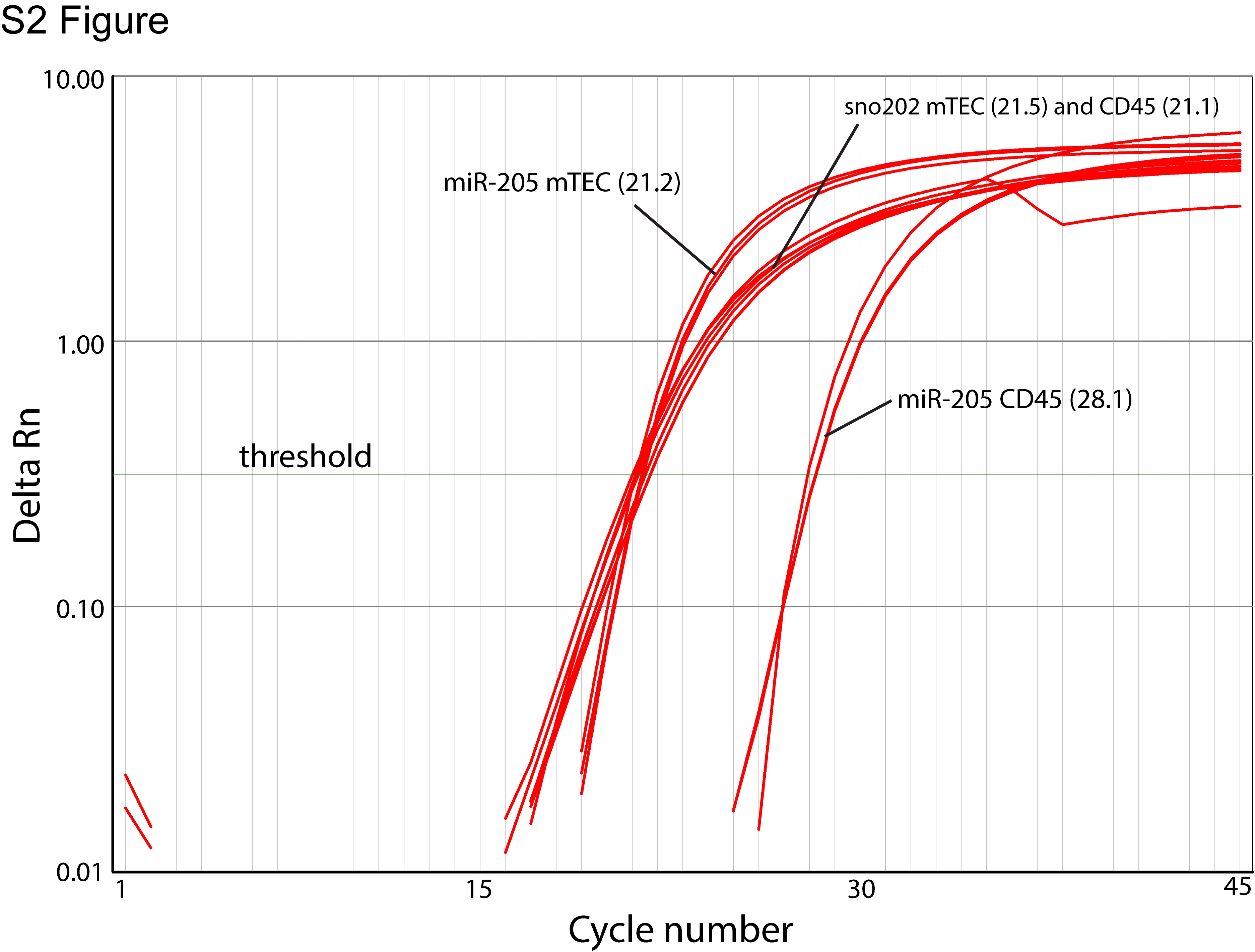

Supplement: S2 Fig — Thymic stromal subsets were purified by flow cytometry from 4–6 week old C57BL/6J wildtype mice to confirm the expression of miR-205 in mTECs by qPCR analysis. Amplification plots are shown for miR-205 and sno202 (internal reference gene) in mTECs and CD45+ cells. The amplification threshold is indicated in green and the threshold cycle for each probe and cell population is indicated in parentheses. (TIF) [file pone.0135440.s002.tif]

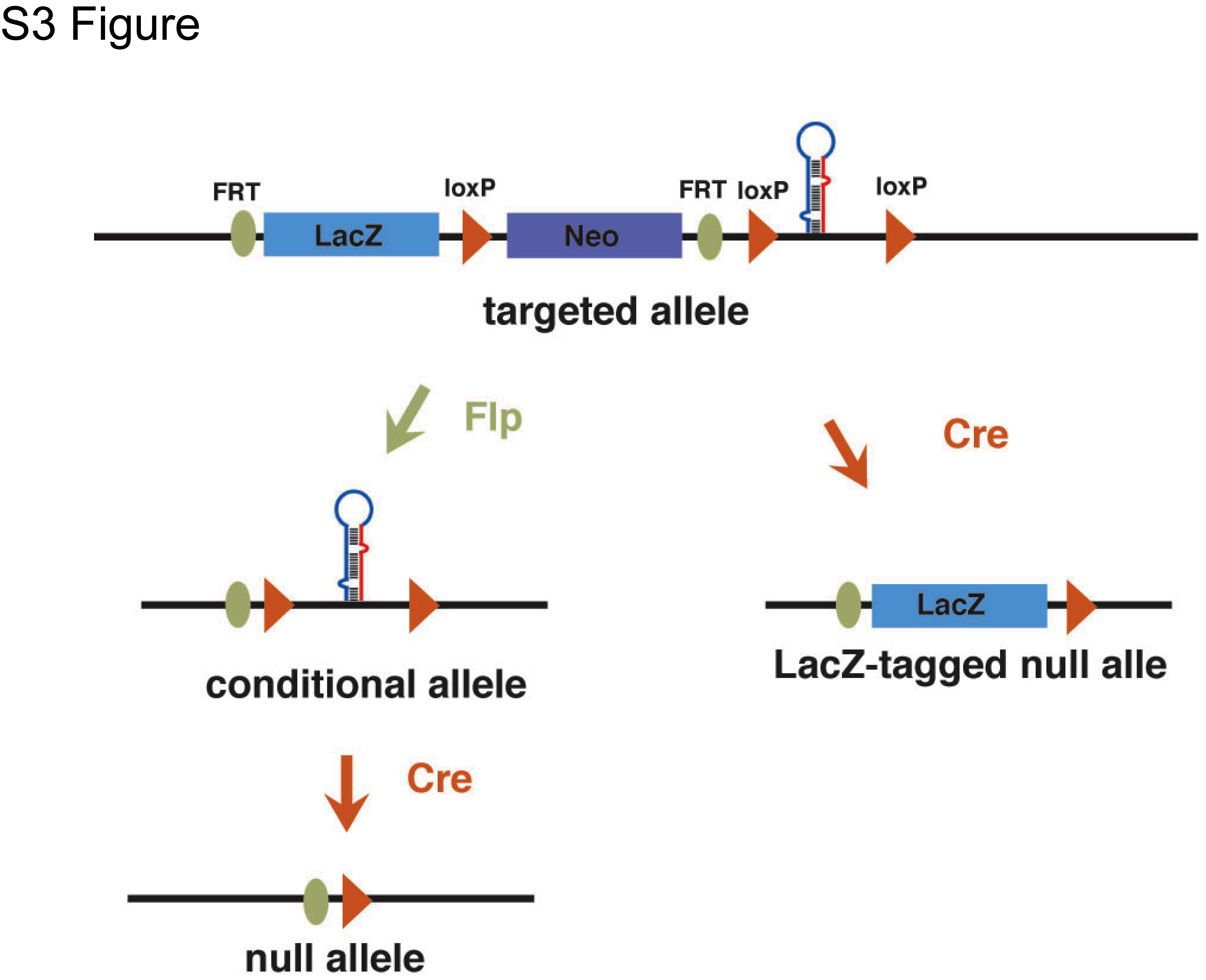

Supplement: S3 Fig — The endogenous miR-205 locus was targeted with a construct containing both a promoter-less lacZ reporter as well as a neomycin cassette. To generate conditional knockout mice, targeted mice were crossed to Rosa26-Flp mice (miR-205 fl/fl), and then bred to FoxN1-Cre mice to ablate miR-205 in TECs (miR-205 ΔTEC). (TIF) [file pone.0135440.s003.tif]
